# Application of tuned liquid column ball damper (TLCBD) for improved vibration control performance of multi-storey structure

**DOI:** 10.1371/journal.pone.0224436

**Published:** 2019-10-24

**Authors:** Muhammad Tanveer, Muhammad Usman, Imdad Ullah Khan, Shakil Ahmad, Asad Hanif, Syed Hassan Farooq

**Affiliations:** 1 School of Civil & Environmental Engineering, National University of Science and Technology (NUST), Sector H-12, Islamabad, Pakistan; 2 Institute of Applied Physics and Materials Engineering, University of Macau, Avenida da Universidade, Taipa, Macau S.A.R China; University of Vigo, SPAIN

## Abstract

Tuned liquid column ball damper (TLCBD) is a passive control device used for controlling the building vibrations induced from wind or earthquakes. TLCBD is a modified form of conventional tuned liquid column damper (TLCD). This paper studies the effect of TLCBD on the four-storey steel frame structure. The performance of the TLCBD is also compared with conventional TLCD. The analytical model of both TLCD and TLCBD is presented here. The effectiveness of these analytical models is examined experimentally by series of shaking table tests under different excitation levels including harmonic loadings and seismic excitations. In TLCBD, the vibration is reduced significantly as compared to TLCD by using steel ball as a moving orifice. The difference in diameter of steel ball and tube, containing the liquid column, acts as an orifice which moves with the movement of the ball. This moving orifice phenomenon enhanced the vibration reduction effect by resisting the water motion in the TLCBD. Root mean square (RMS) and peak values of acceleration were calculated for each loading and each storey of uncontrolled and controlled structures. Comparison of the time histories of controlled and uncontrolled structures for different loadings is also reported. Results indicate that the TLCBD is more effective in the earthquake scenarios as compared to the harmonic excitations. The TLCBD controls the vibration of the primary structure significantly in vibration reduction.

## Introduction

Due to the persistently increasing trend in construction of tall buildings and using lightweight aggregates as well as high strength materials, the tall building structures are susceptible to greater vibrations and deflections under wind loads and seismic excitations [1]. With increasing height of the structure, it becomes more sensitive to wind loads and earthquake loadings. In order to control the structures from these vibrations, different dampers and isolators have been proposed by researchers. These include base isolator, tuned mass damper (TMD) and tuned liquid column damper (TLCD)[2–4]. Among these is the TLCD which was initially proposed by Sakai in 1989 [5].

TLCD has a wide range of applications, and has been designed especially for tall buildings and towers [5,6]. TLCD is a passive damper, which includes a U-shaped tubular component having a liquid inside it. It has an orifice which dissipates the energy from the structure during vibration. Many studies have been carried out earlier to optimize and improve the performance of TLCD. Different techniques were used to improve the damping of TLCD by using high viscous liquid, variable orifice and changing the shape of water container [7–9]. At first, the optimization of TLCD was done by obtaining optimum tuning ratio and optimum damping ratio, for structures subjected to wind and earthquake loadings [7]. Later, more viscous liquids have also been studied to improve the damping effect of TLD in low rise reinforced concrete frame buildings [8]. Different shapes of TLCD, such as U-shaped cross-sections, V-shaped cross sections, and spherical cross-section areas, have also been studied [10]. V-shaped TLCD has been proven to be more effective than U-shaped TLCD in suppressing strong wind vibrations [9]. TLCD has also been tested for the control performance against lateral and harmonic vibrations [11,12]. The circular shaped TLCD has shown better effectiveness against torsional vibrations than the conventional TLCD [13]. Water used in the TLCD can be further utilized for other purposes like water supply and firefighting [9]. The concept of multiple tuned liquid column dampers (MTLCD) has also been introduced in previous studies [14]. The effect of MTLCD has been studied on high rise building and bridges. Design parameters, such as tuning ratio and damping ratio have been derived against seismic loading [14]. MTLCD has been more efficient than single TLCD in reducing the structural responses against vibration [15,16]. Semi-active TLCD has also been designed for improved vibrations control. Semi-active TLCD showed the best features of both passive and active TLCD [17,18], thus it is economical to design semi-active rather than an active damper.

For performance enhancement, different semi-active algorithms based on fuzzy control strategy have been developed and compared with those for passive TLCD. Magneto-rheological (MR) liquid in semi-active TLCD has been more effective than conventional TLCD in the vibration control of tall buildings [19,20]. The performance of TLCD has been also enhanced by providing imprints to the walls of the TLCD that increased the damping effect of the conventional TLCD [21]. A compliant TLD has been designed which also enhanced the performance of conventional TLD for controlling seismic vibrations of short period structure [22]. MR elastomers have also been utilized in the smart base isolation system in the building structures [23,24]. Optimization of semi-active TLCD for non-linear multi-degree of freedom (MDOF) systems by statistical linearization method has further improved the effectiveness in tall buildings [25]. Several studies have been conducted on TLCD for base isolated structures, that can be referred as a hybrid system [26,27]. Optimal design for base-isolated structure TLCD has been proposed for seismic loadings, which significantly reduced the overall vibration of the structure significantly [28]. The effect of near and far field earthquakes were also studied on different base isolation techniques. The dynamic response of the base isolated structure depends on the source of excitation [29].

Recently, some studies have been carried out by replacing the orifice of TLCD by moving steel ball in the horizontal portion of TLCD, which was subsequently called tuned liquid column ball damper (TLCBD) [30,31]. The performance of TLCBD has been largely dependent on the tuning ratio and ball to tube diameter ratio [31]. The damping in the TLCBD has been considered to be linear which has been proven by the free vibration test [30]. The function of the ball has been the same as moving orifice (i.e. to reduce the water displacement in TLCBD) [30]. For performance improvement of TLCBD, the ball was attached to the spring inside the horizontal portion of TLCBD [32]. Ball in the circular TLCD as a moving orifice has been also used to control torsionally coupled vibrations [33].

TLCBD has been previously designed and tested for single degree of freedom SDOF structure only [30,31]. In this particular study, TLCBD has been designed and tested for the multi degrees of freedom MDOF structure which has never been studied previously. The subsequent effect of TLCBD on the response of each storey of the structure was determined. For the performance comparison, the response of TLCBD is compared with the response of the TLCD and uncontrolled primary structure. For time history analysis, controlled structures having TLCBD, TLCD and uncontrolled structure have been subjected to base excitation which included some historical earthquake data and the harmonic vibrations. The acceleration response of each storey against each loading has been calculated and analyzed. The peak and RMS acceleration of controlled and uncontrolled structure have also been compared. The detailed experimental testing has been also performed by using a shake table in the laboratory.

## Analytical model

### System description

**Fig 1:**
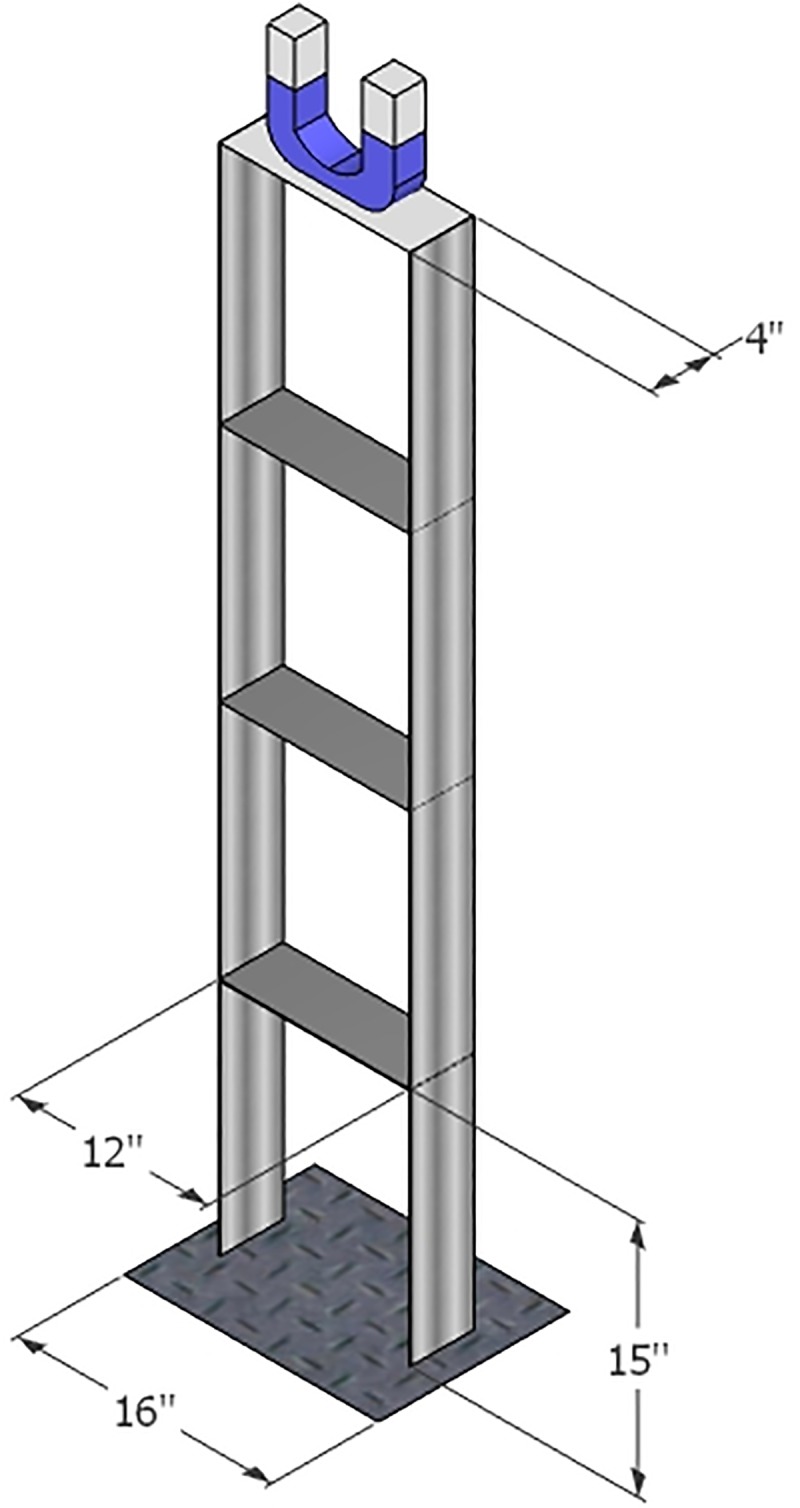
Schematic of a multi-storey structure model equipped with TLCBD.

The description of MDOF system having TLCBD on the top storey under different seismic loading and harmonic excitations is shown in Fig 1. Building model is made up of steel frame and has 4 storeys (each of 15 inches height). The length of the beam for each storey is 12 inch. The width of the beam and column for each story is 4 inch. The building model has been adopted from Inamdar et.al [34]. The other parameters including mass, stiffness and damping of the primary structure are listed in Table 1

**Table 1:** Primary structure parameters.

Storey level	Mass (Kg)	Stiffness (N/m)	Damping (Nsec/m)	Modes	Natural Frequency (rad/sec)
1	2.2	1505.10	1.720	First Mode	9.08
2	2.2	1505.10	1.720	Second Mode	26.15
3	2.2	1505.10	1.720	Third Mode	40.07
4	2.2	1505.10	1.720	Fourth Mode	49.15

As the TLCD or TLCBD is designed for the specified fundamental mode of vibration [30], in this study TLCBD is designed for the first fundamental mode of vibration. TLCBD is a U-shaped tube which is filled with liquid. A spherical ball is placed in the horizontal portion of the tube acting like moving orifice that moves in the horizontal portion of the tube during vibration in the liquid. The function of this ball has to reduce the motion of the liquid in TLCBD [30,31]. TLCBD is 2 DOF system having 1 DOF each for liquid and ball. The equation of motion for the ball is derived by using Lagrange derivations [30] which can be expressed as:

$$\left(m_{b}+\frac{J_{b}}{R_{b}^{2}}\right)\ddot{x_{b}}+d_{eq}\dot{x_{b}}=\left(\frac{J_{b}}{R_{b}^{2}}\right)\ddot{{(x}_{s}}+{\ddot{x}}_{g})+d_{eq}{\dot{x}}_{l}+\left(\frac{2m_{l}gR_{bt}^{2}}{L_{t}}\right)x_{l}$$ (1)

In the above equation *m*_*b*_ is a mass of the ball, *d*_*eq*_ is the equivalent damping coefficient of the ball in the liquid, *m*_*l*_ is the liquid mass, *J*_*b*_ is the mass moment of inertia about its center of mass, *R*_*b*_ is the radius of ball, *R*_*bt*_ is the ball-to-tube diameter ratio, *g* is the gravitational acceleration and *L*_*t*_ is the total length of the tube which is equal to (*L*_*t*_ = *L*_*h*_+2*L*_*v*_), where *L*_*h*_ is the horizontal length and *L*_*v*_ is the vertical length of the tube. Also

$$J_{b}=\frac{2m_{b}R_{b}^{2}}{5}$$ (2)

$$d_{eq}=6\pi \nu R_{b}$$ (3)

In Eq (3), ν is the kinematic viscosity of the liquid used in the tube.

The equation of motion for liquid was also derived by Lagrange derivation [30] and can be expressed as:

$$m_{l}\ddot{x_{l}}+(2m_{l}\xi_{l}\omega_{d})\dot{x_{l}}+\left(\frac{2m_{l}g}{L_{t}}\right)x_{l}=-\rho m_{l}(\ddot{x_{s}}+\ddot{x_{g}})$$ (4)

In Eq (4) *ω*_*d*_ is the frequency of the liquid in the tube, *ξ*_*l*_ is head loss coefficient of the liquid in the tube,. This value depends on the ball-to-tube diameter ratio *R*_*bt*_ and was calculated against different *R*_*bt*_ ratios [30]. ρ is the length ratio parameter and is equal to length of the horizontal length *L*_*h*_ to total length *L*_*t*_ of the tube. (*ρ* = *L*_*h*_/*L*_*t*_).

### The equation of motion for MDOF system equipped with TLCBD

Some previous studies were done on TLCBD which was equipped with an SDOF system [30,31]. In this research, TLCBD has been designed for MDOF structure. The coupled equation of motions of structure equipped with TLCBD is given as Eq (5):

$$\left(m_{4}+m_{l}+\frac{J_{b}}{R_{b}^{2}}\right)\ddot{x_{4}}+c_{4}\dot{x_{4}}+k_{4}x_{4}=-\left(m_{4}+m_{l}+\frac{J_{b}}{R_{b}^{2}}\right)\ddot{x_{g}}-\rho m_{l}\ddot{x_{l}}+\left(\frac{J_{b}}{R_{b}^{2}}\right)\ddot{x_{b}}$$ (5)

The equations of motion can be presented in the form matrices for MDOF-TLCBD combined system. The matrices of the mass, stiffness and damping of the combined system are given in Eqs (6) through (8).

$$M=\left[\begin{array}{cccccc} m_{1} & 0 & 0 & 0 & 0 & 0\\ 0 & m_{2} & 0 & 0 & 0 & 0\\ 0 & 0 & m_{3} & 0 & 0 & 0\\ 0 & 0 & 0 & M_{4} & pml & -m_{bb}\\ 0 & 0 & 0 & pml & m_{l} & 0\\ 0 & 0 & 0 & -m_{bb} & 0 & M_{b} \end{array}\right]$$ (6)

In Eq (6), ‘M’ is the mass matrix of the controlled structure. m_1_, m_2_ and m_3_ are mass of each storey 1, 2 and 3 respectively. M_4_ is the mass of 4^th^ storey combined with TLCBD and equal to $m_{4}+m_{l}+\frac{J_{b}}{R_{b}^{2}},\;m_{bb}=\frac{J_{b}}{R_{b}^{2}}$ and $M_{b}=m_{b}+\frac{J_{b}}{R_{b}^{2}}$ which is the total mass of the ball.

$$K=\left[\begin{array}{cccccc} k_{1}+k_{2} & -k_{2} & 0 & 0 & 0 & 0\\ -k_{2} & k_{2}+k_{3} & -k_{3} & 0 & 0 & 0\\ 0 & -k_{3} & k_{3}+k_{4} & -k_{4} & 0 & 0\\ 0 & 0 & -k_{4} & k_{4} & 0 & 0\\ 0 & 0 & 0 & 0 & k_{L} & 0\\ 0 & 0 & 0 & 0 & -K_{L} & 0 \end{array}\right]$$ (7)

In Eq (7) ‘K’ is the stiffness matrix of the controlled structure. *k*_1_, *k*_2_, *k*_3_ and *k*_4_ are the stiffness of each storey 1, 2, 3 and 4 respectively. $k_{L}=\frac{2m_{l}g}{L_{t}}$ is the stiffness of the liquid and $K_{L}=\frac{2m_{l}gR_{bt}^{2}}{L_{t}}$ is the stiffness of the liquid in ball equation.

$$C=\left[\begin{array}{cccccc} c_{1}+c_{2} & -c_{2} & 0 & 0 & 0 & 0\\ -c_{2} & c_{2}+c_{3} & -c_{3} & 0 & 0 & 0\\ 0 & -c_{3} & c_{3}+c_{4} & -c_{4} & 0 & 0\\ 0 & 0 & -c_{4} & c_{4} & 0 & 0\\ 0 & 0 & 0 & 0 & c_{L} & 0\\ 0 & 0 & 0 & 0 & -d_{eq} & d_{eq} \end{array}\right]$$ (8)

In Eq (8) ‘C’ is the stiffness matrix of the controlled structure. *c*_1_, *c*_2_, *c*_3_ and *c*_4_ are the damping of each storey 1, 2, 3 and 4 respectively. *c*_*L*_ = 2*m*_*l*_*ξ*_*l*_*ω*_*d*_ is the damping of the liquid in TLCBD and *d*_*eq*_ is the equivalent damping coefficient of the ball.

The above MDOF-TLCBD has 6 DOF having 1 DOF for each storey and 2 DOF for TLCBD. It can be expressed in generalized matrix form as Eq 9:

$${\left[M\right]}_{6*6}\{\ddot{x}\}+{\left[C\right]}_{6*6}\{\dot{x}\}+{\left[K\right]}_{6*6}\{x\}={-[M]}_{6*6}\{z\}\ddot{x_{g}}$$ (9)

In Eq (9), [*M*], [*C*] and [*K*] are mass, damping and stiffness matrix respectively having order 6*6. $\left\{\ddot{x}\right\}$, $\{\dot{x}\},$ {*x*} are acceleration, velocity and displacement vectors. The displacement vector can be expressed as {*x*} = {*x*_1_,*x*_2_,*x*_3_,*x*_4_,*x*_*l*_,*x*_*b*_}^*T*^. Same can be expressed for acceleration and velocity. {*z*} is the influence coefficient vector and can be define as {*z*} = {1,1,1,1,0,0}.

### Design of TLCBD model

**Fig 2:**
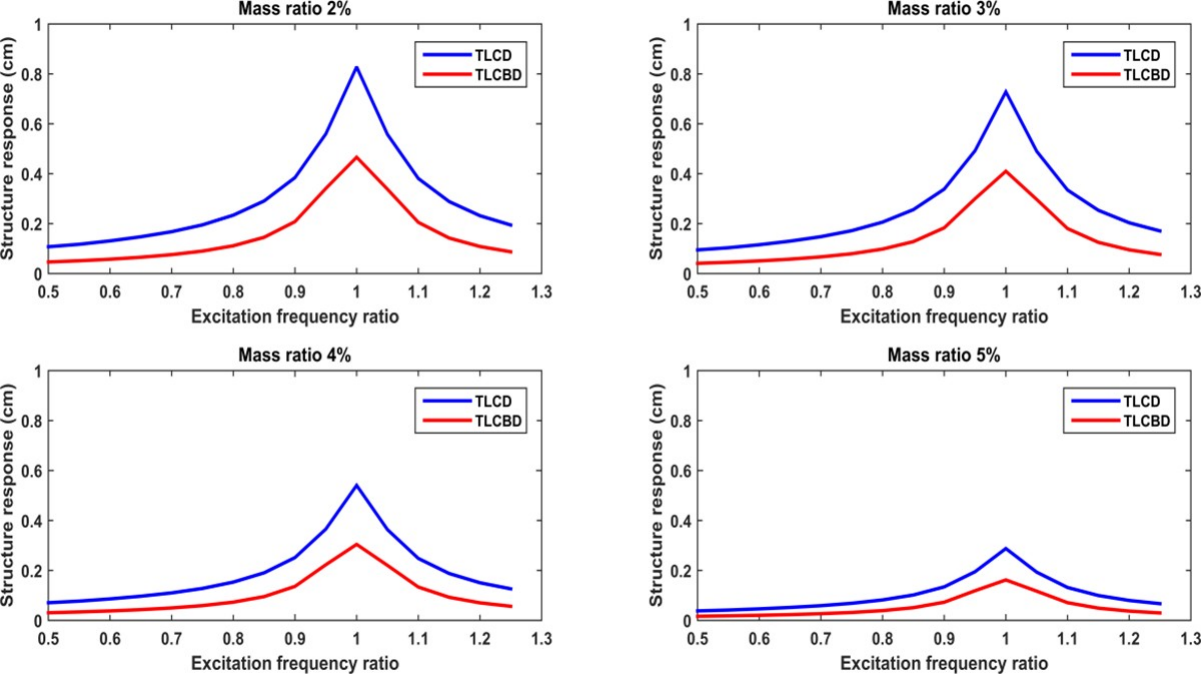
Variation of displacement of structure with various excitation frequency ratios and mass ratios.

Based on the aforementioned equations, the analytical model of the TLCBD has been designed. For the design optimization, the performance of the both TLCD and TLCBD has been analyzed using different mass ratios ranges from 2% to 5%. Mass ratio can be defined as the ratio between mass of the damper to the mass of the primary structure. Different excitation frequency ratios ranges from 0.5 to 1.2 have been applied for performance evaluation. Excitation frequency ratio can be defined as the ratio between external excitation frequencies to the natural frequency of the structure. The length ratio *ρ* and tuning ratio for both TLCBD and TLCD have been adopted from the literature as 0.7 and 0.97 respectively [30,31]. The ball-to-tube diameter ratio *R*_*bt*_ for TLCBD was optimized as 0.8 [30]. The natural frequency of the structure model is 9.08 rad/sec (1.45 Hz). Fig 2 shows the response comparison of TLCD and TLCBD. At excitation frequency ratio ranges from 0.5 to 0.7, no significant difference has been observed in the response of TLCD and TLCBD. TLCBD reduced the response of the building significantly as the excitation frequency ratio increases from 0.7 to 0.9, which is close to resonance region. In SDOF system, the structure response is almost similar for both TLCD and TLCBD for frequency ratios in the range of 0.7 to 0.9 [30]. However, in the case of MDOF system, the performance of the TLCBD has been observed to be better than TLCD. The performance of the TLCBD was much better for the frequency ratios near 1, which may be called the resonance region. At resonance region, when the excitation frequency is equal to natural frequency of structure the response of structure becomes maximum [35]. The performance of the TLCBD also depends on the mass ratio. From Fig 2 it can be clearly seen that in all mass ratios, the TLCBD response has been better than TLCD. Increasing the mass ratio will improve the performance of the controlled structure. However increasing the mass ratio will increase the mass of the TLCBD or other absorber causing high cost. Therefore, it should be better to choose the practical mass ratio depending on the weight of the primary structure and application of the absorber.

Based on the analytical model results, the parameters of the TLCBD and TLCD model are listed in the Table 2, which are subsequently used for developing the experimental model. For comparison purpose, all parameters of the TLCBD and TLCD have been kept the same.

**Table 2:** Design parameters of TLCBD and TLCD model.

Parameters	TLCBD	TLCD
Mass ratio	5%	5%
Tuning ratio	0.97	0.97
Length ratio (*ρ*)	0.7	0.7
Density of liquid (Kg/m^3^)	1000	1000
Density of ball (Kg/m^3^)	7500	
Ball-to-tube diameter ratio (*R*_*bt*_)	0.8	
Kinematic viscosity (Nm/sec)	0.001	
External frequency ratio	0.5, 0.6, 0.7, 0.8, 0.9, 1, 1.1, 1.2	0.5, 0.6, 0.7, 0.8, 0.9, 1, 1.1, 1.2

Following steps have been recommended by Al Saif et.al and Gur.et al [30,31] to design the SDOF-TLCBD. The same procedure has been adopted for the design of TLCBD for the MDOF structure.

1. The fundamental natural frequency of the primary structure has been obtained analytically using MATLAB. By taking the tuning ratio 0.97 the frequency of TLCBD has been obtained.
2. The mass of the liquid has been calculated by selecting the mass ratio of 5%.
3. The length of the TLCBD has been obtained from the frequency of the TLCBD using the relation (wd^2^ = 2g/L_t_).
4. By using a length ratio of usually 0.7, a length of vertical and horizontal columns has been obtained.
5. From the obtained mass of the liquid, the volume of the TLCBD and diameter of the tube has been calculated.
6. From the diameter of the tube, the ball diameter has been obtained by using the ball to tube diameter ratio in the range of 0.7 to 0.8.

## Experimental validation

To validate the analytical model, the experimental model of both TLCD and TLCBD has been fabricated in the laboratory. Different harmonic and earthquake excitations have been applied to study the dynamic behavior of structure equipped with TLCD and TLCBD using shake table.

### Experimental setup

**Fig 3:**
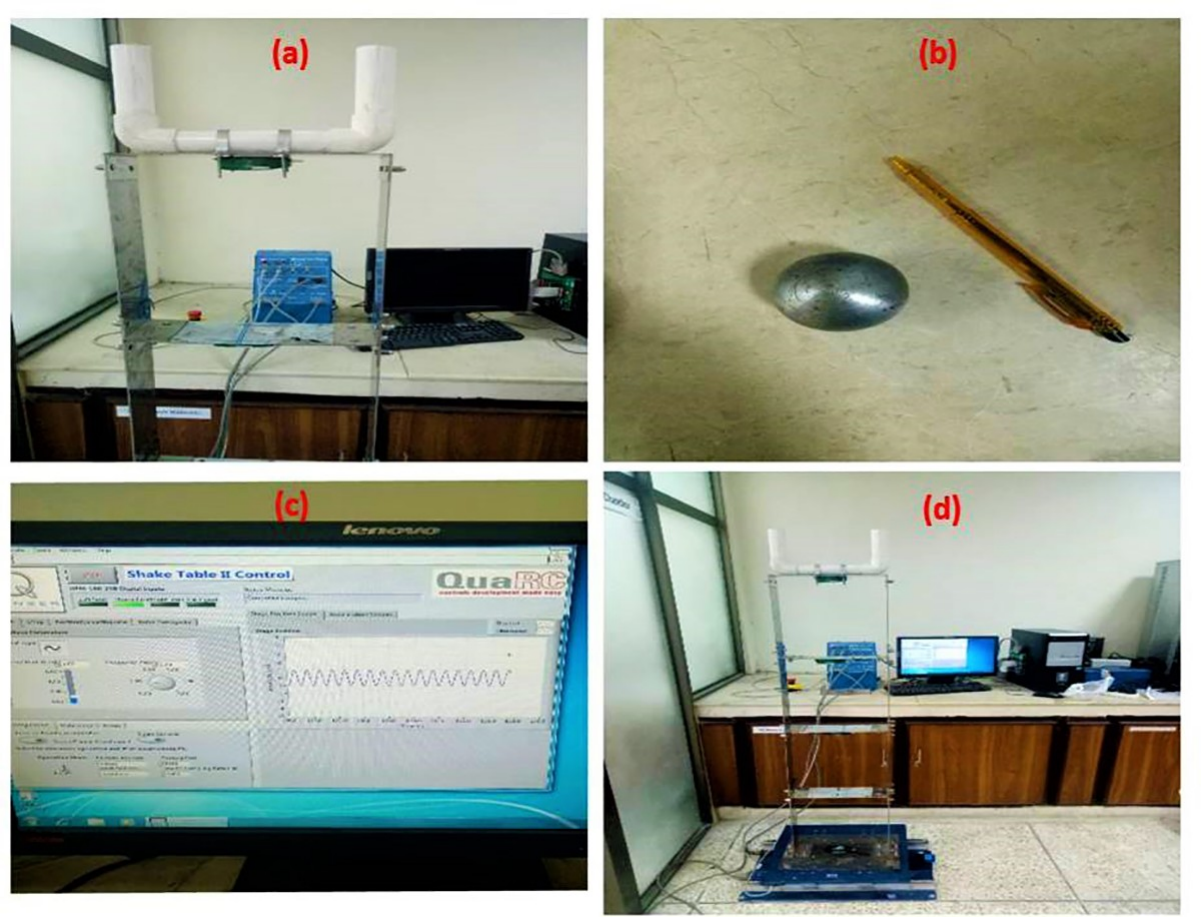
(a) TLCBD model; (b) Spherical steel ball; (c) QuaRC software window; (d) All experimental setup.

Experimental setup included a steel frame, TLCBD, TLCD and shake table, as shown in Fig 3. Primary structure has been fabricated of stainless steel and consists of 4 storeys. Each storey has the same height and mass. The steel frame was fixed on a base plate having length 14 inches. Holes were drilled on the base plate to fix the base plate on the top stage of the shake table. Both TLCD and TLCBD have been tuned according to the first natural frequency of the model, and have been have been placed on the 4^th^ storey of the structure. Normal water has been used in the U-shaped TLCBD, while the steel ball was placed in the middle of the horizontal section of the TLCBD (Fig 3B).

Shake table used for the experiments is unidirectional, and operated by a powerful brushless motor. The maximum payload of the table is 15 kilogram which can achieve a maximum acceleration of 2.5g. The mass of the total shaking table system is 27.2 kilogram. Dimensions of the top stage are 0.46 x 0.46 m^2^ and the bottom stage is 0.61 x 0.46 m^2^. The shake table was operated by using QuaRC software, as shown in Fig 3C. Accelerometers X2-02 have been used for recording the acceleration data during excitations. The accelerometers were attached on the base of each storey using double tape as shown in Fig 3A. The data was saved in the accelerometers in the form of a CSV file. The pre-processing of the accelerometers was done before starting the experiments [36]. The entire system consisted of a universal power module (UPM), accelerometers attached to each storey of the structure, a PC running the control software, and shaking table as shown in Fig 3D.

For response analysis, MDOF-TLCBD and TLCD structure have been subjected to different types of loadings. Harmonic loading having frequency 0.5 Hz, 1 Hz and 1.5 Hz respectively along with sine sweep wave having 0.15 cm amplitude. The earthquake loading was also applied by shake table. These included earthquake loadings of Northridge and Kobe. Time history analysis was also done for primary structure, TLCBD and TLCD structure.

### Experimental results and discussions

#### Description of results

For response analysis, the acceleration responses of the MDOF-TLCBD model were recorded against different loading conditions. Table 3 shows the comparison of the response of the TLCBD, TLCD and uncontrolled structure against different excitations. The peak and root mean square (RMS) values of acceleration against each excitation at every storey level were calculated. The peak accelerations show the maximum acceleration response value of structure against different excitation. The RMS values indicate the vibration energy of the structure against respective loading. Regarding vibration reduction, the results indicated that TLCBD reduced the response of the structure better than TLCD. This signifies that TLCBD is more effective in the dissipating the vibration energy and providing adequate damping to the structure.

**Table 3:** Peak and RMS acceleration (g) value of TLCD, TLCBD and uncontrolled structure.

Story level	Cases	0.5 Hz		1 Hz		1.5 Hz		Sine Sweep		Northridge		Kobe	
		Peak	RMS	Peak	RMS	Peak	RMS	Peak	RMS	Peak	RMS	Peak	RMS
1	UC	0.391	0.103	0.388	0.098	0.514	0.102	0.523	0.093	0.494	0.085	0.288	0.067
	TLCD	0.321	0.085	0.303	0.077	0.427	0.085	0.450	0.083	0.385	0.067	0.242	0.056
	TLCBD	0.290	0.078	0.275	0.069	0.395	0.082	0.425	0.080	0.362	0.061	0.296	0.053
2	UC	0.573	0.123	0.478	0.120	0.746	0.190	0.559	0.122	0.583	0.130	0.624	0.097
	TLCD	0.458	0.099	0.407	0.102	0.597	0.152	0.475	0.104	0.379	0.084	0.499	0.078
	TLCBD	0.364	0.100	0.490	0.100	0.486	0.124	0.330	0.097	0.325	0.058	0.462	0.072
3	UC	0.552	0.121	0.390	0.103	0.549	0.142	0.583	0.122	0.672	0.157	0.588	0.104
	TLCD	0.430	0.094	0.320	0.085	0.489	0.127	0.437	0.094	0.430	0.101	0.441	0.079
	TLCBD	0.323	0.084	0.305	0.077	0.550	0.122	0.378	0.081	0.448	0.060	0.529	0.072
4	UC	0.858	0.198	0.660	0.163	0.930	0.243	0.773	0.182	1.036	0.214	0.871	0.148
	TLCD	0.626	0.145	0.449	0.111	0.661	0.176	0.495	0.118	0.622	0.128	0.592	0.100
	TLCBD	0.538	0.113	0.332	0.080	0.512	0.134	0.341	0.090	0.429	0.070	0.520	0.085

The RMS and peak acceleration response of TLCBD controlled structure is better than corresponding response of uncontrolled and TLCD controlled structure. However, in some cases, the peak acceleration of the TLCBD controlled structure was found higher than TLCD controlled structure. The response of the controlled structure in reducing vibrations is different under each loading parameter. In all excitations, the 4^th^ storey of the uncontrolled structure showed maximum response against each loading. With the increase in frequency of excitation, the wave amplitude also increases and consequently phenomenon of response reduction of the TLCD or TLCBD also improved [8]. TLCBD was placed on the 4^th^ storey of building model, which reduced the response of the top storey. As discussed, the analytical model of TLCBD reduced the response of the building model better than TLCD in all applied excitation frequencies. The result of experimental model also proves the better performance of the TLCBD over TLCD.

For a better understanding of results, percentage reduction of RMS acceleration with average has been calculated for each storey (Table 4). Similar trend has been observed for both TLCD and TLCBD in each storey against all loadings. However, it could be seen that the response of TLCBD was much better than TLCD. TLCBD performed better because some of the input energy provided to the primary structure is dissipated by the ball in the form of angular and kinetic energies. Moreover, some part of the input energy is also dissipated due to friction between ball and liquid in the TLCBD [30], which was included in the Eq (1) of the ball in form of ′*d*_*eq*_′. Thus in the TLCBD, we have two dynamic absorbers in term of liquid and ball added to the primary structure [30]. However in, TLCD only liquid dissipates the energy from the primary structure.

**Table 4:** Percentage reduction of RMS acceleration (g).

No. of story	Cases	0.5 Hz	1 Hz	1.5 Hz	Sine Sweep	Northridge	Kobe	Average
1	TLCBD	24.30	29.56	19.65	13.92	28.71	20.54	22.78
	TLCD	18.01	21.81	16.91	10.71	21.76	15.59	17.50
2	TLCBD	19.04	16.64	34.84	20.77	55.01	25.75	28.68
	TLCD	19.77	14.55	19.98	14.45	34.98	19.44	20.50
3	TLCBD	30.68	25.94	13.96	33.69	61.88	31.06	32.87
	TLCD	21.89	18.10	10.79	23.14	35.63	24.52	22.30
4	TLCBD	42.89	51.04	44.69	50.33	67.26	42.58	49.80
	TLCD	26.78	32.00	27.35	35.44	39.86	31.93	32.20

Under seismic excitations, TLCBD showed better performance and high vibration reduction especially in the case of Northridge earthquake response as compare to Kobe earthquake response. As shown in Table 4, the maximum response reduction of 67.26% has been observed in Northridge at 4^th^ storey among all loadings. In sine sweep, Northridge and Kobe excitations, the response reduction increases from 1^st^ to top storey level. This trend was not followed in harmonic loading as the frequency of each storey was changed. Under harmonic loading, maximum percentage reduction has been observed for 1 Hz at the 4^th^ storey and 1^st^ storey. At 2^nd^ storey, TLCBD showed better performance for 1.5 Hz frequency, while at the 3^rd^ storey, the maximum reduction was observed for 0.5 Hz frequency case. Regarding response reduction of RMS acceleration similar trend was observed. The response reduction in case of the TLCBD has been better than TLCD case except at 2^nd^ storey against 0.5Hz. Average percentage reduction of RMS acceleration of each storey has been also calculated. The maximum average percentage reduction has been reported as 49.80% at the top storey of the primary structure. The average reduction has been also increased from 1^st^ storey to top storey of the primary structure.

**Fig 4:**
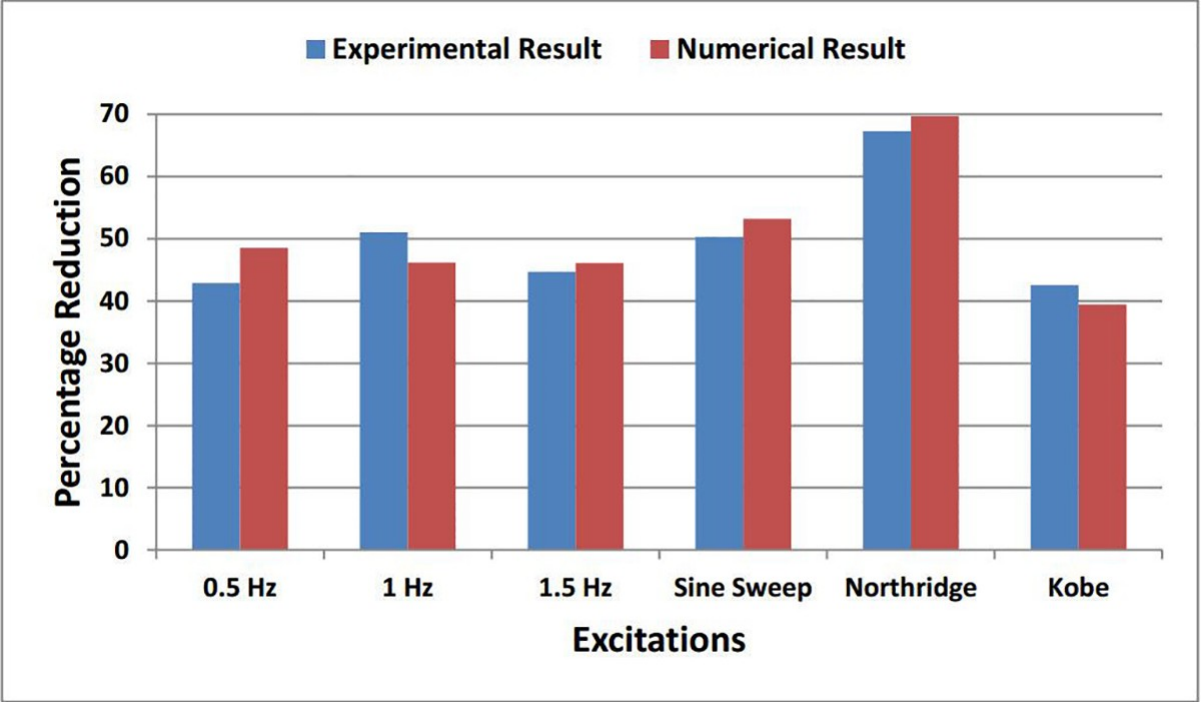
Comparison of experimental and numerical RMS acceleration percentage reduction.

Fig 4 shows the comparison of the RMS acceleration percentage reduction between numerical and experimental results of top storey. The comparison has been made of TLCBD controlled structure against each loading including harmonic and earthquake excitations. The overall differences of response reduction are within the reasonable range between experimental results and numerical study. In addition both numerical and experimental results showed significant response reduction of controlled structure having TLCBD on top storey.

### Storey level response comparison

**Fig 5:**
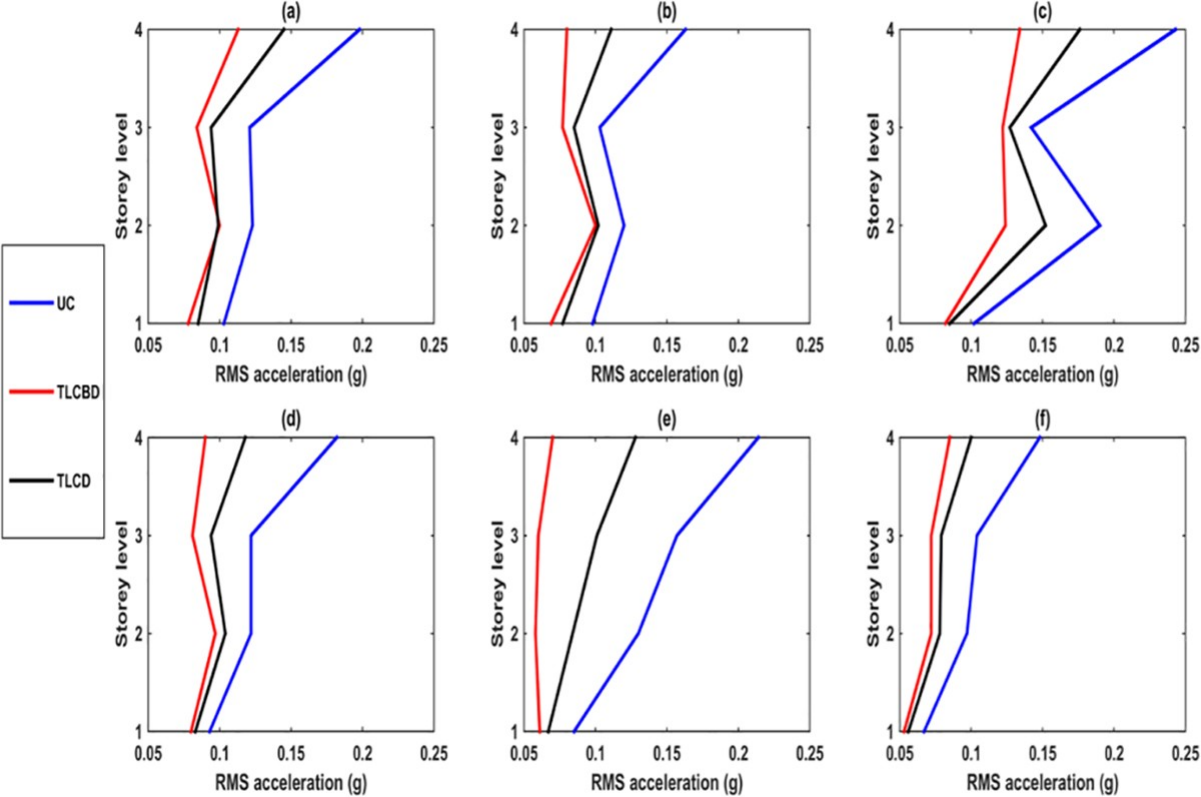
RMS acceleration responses comparison of TLCBD, TLCD and Uncontrolled structure: (a) 0.5Hz; (b) 1Hz; (c) 1.5Hz; (d) Sine sweep; (e) Northridge; and (f) Kobe.

Fig 5 shows the comparison of TLCBD, TLCD and uncontrolled RMS acceleration of each storey level. As shown in each loading, TLCBD controls the vibration of structure better than TLCD, against each loading. At 1^st^ storey, the difference in the response between TLCD and TLCBD was observed less than the response of other storeys. It was also observed that the response of 2^nd^ storey was greater than that for 3^rd^ storey (for both controlled and uncontrolled structure in each loading except the Northridge earthquake). This trend was primarily seen due to low column stiffness of the proposed structural model. The minimum uncontrolled response of the model was observed in the Kobe earthquake on the top storey, whereas the maximum uncontrolled response was observed in 1.5Hz loading on the top storey, as this loading frequency lies in the resonance region. In all loadings, maximum response reduction was observed on the top storey, as the uncontrolled structure showed a maximum response on a top storey level. Further, TLCBD showed better performance in vibration control in Northridge earthquake among all loadings, because as the excitation frequency increased the water motion increased in liquid damper, thereby making the damper become more effective in vibration reduction [8].

#### Time history analysis

**Fig 6:**
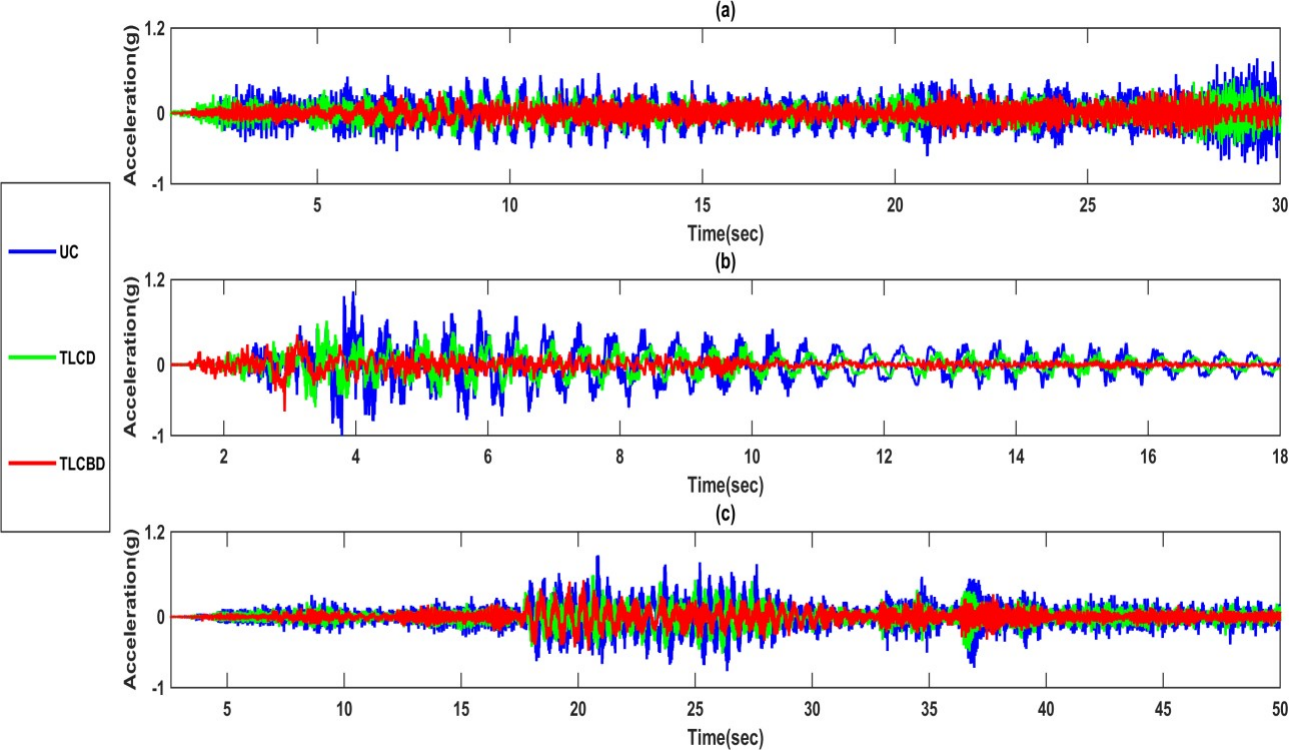
Time histories response comparison of TLCBD, TLCD and Uncontrolled structure: (a) Sine sweep; (b) Northridge; (c) Kobe.

The comparison has been made between time histories of TLCD, TLCBD and uncontrolled structure. Fig 6 showed the Northridge, Kobe and Sine sweep acceleration time histories of the top storey. It has been observed that the response was reduced considerably in the case of TLCBD structure as compared to TLCD. In the time history of the Northridge earthquake (Fig 6B), TLCBD clearly showed reduced response and peaks of the primary structure. On the other hand, in the Kobe earthquake (Fig 6C) the response reduction is considerably less than the Northridge earthquake. The peaks were reduced but not significantly as were seen for the case of Northridge. This phenomenon is common in TLCD as well due to the sudden abrupt change in the amplitude and frequency of the Kobe earthquake. In sine sweep wave, the amplitude remained constant and frequency gets increasing after some interval of time. Regarding response reduction in sine sweep case (Fig 6A), the TLCBD controlled structure showed the reduced response and peaks in high-frequency range of sine sweep wave significantly as compared to the low-frequency range of the sine sweep wave.

**Fig 7:**
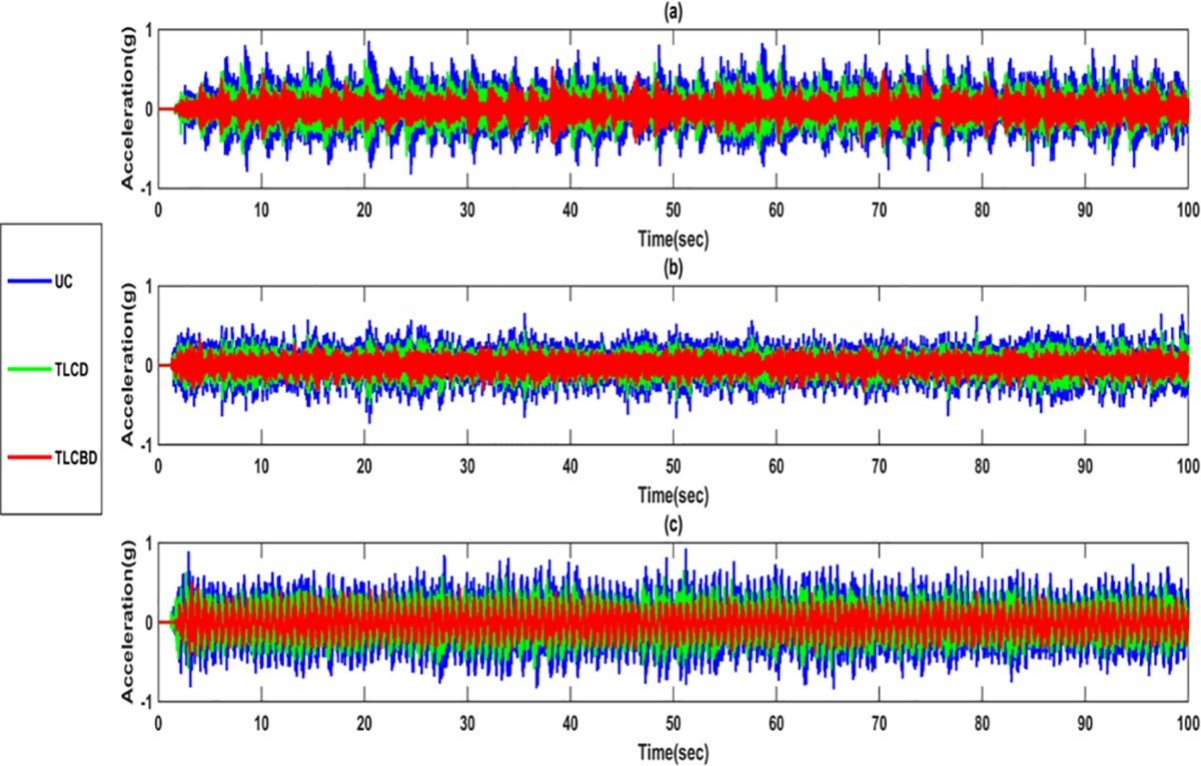
Time histories response comparison of TLCBD, TLCD and Uncontrolled structure: (a) 0.5Hz; (b) 1Hz; (c) 1.5Hz.

Fig 7 shows the time histories of the top storey acceleration of the harmonic loading case. Under harmonic loading, the maximum response reduction was observed for the 1.5 Hz (Fig 7C) significantly. As seen in the time histories of 1Hz (Fig 7B) and 1.5 Hz case (Fig 7C), the response of the TLCBD remains constant during the period of excitation. On the contrary, in 0.5 Hz the peaks were observed after some time interval throughout the excitation period. As discussed in Table 3 and Table 4, it is also clearly seen in time histories analysis of all loadings that TLCBD reduces the response of the primary structure significantly as compared to TLCD

## Conclusions

The present study has focused on the performance of the TLCBD controlled four storey building structure. Analytical models for both TLCD and TLCBD have been presented. Steel ball and normal water have been used in the development of TLCBD. Detailed experimental investigation has been done using shaking table tests. The performance of TLCBD has also been compared with TLCD using the same design parameters for both models. From the analytical and experimental investigation, it was shown that TLCBD was effective in reducing the response and vibration of each storey significantly. It has been observed that response reduction was much more prominent in RMS acceleration response of each storey against different loadings as compared to peak accelerations responses. The response of the controlled structure varied from 1^st^ to top storey, and showed better performance regarding vibration reduction on the top storey of the primary structure. TLCBD performance also exhibited dependence on the loading conditions. Under seismic excitations, the maximum RMS response reduction was observed at the 4^th^ storey for Northridge earthquake case that was 67.26% while under harmonic loading; the maximum reduction was 51.04%. Under the sine sweep loading, the maximum response reduction was 50.33% at the top storey of the structure. The performance of the TLCBD or TLCD is dependent on the magnitude of the excitation frequency that may reduce in the strong excitation or wind loading. Therefore, in the Kobe earthquake case, the response reduction was less compared to the Northridge earthquake case for both TLCBD and TLCD controlled structures. Hence TLCBD showed better performance under seismic excitation having low amplitudes and also significantly reduced the response under the harmonic loading. The similar trend has been observed in the response of the TLCD controlled structure under both seismic excitation and harmonic excitation as in TLCBD. It has been concluded from the comparison of overall response between TLCD and TLCBD that TLCBD reduced the response of each storey significantly and more than TLCD.

## Supporting information

S1 Table (XLSX)

S2 Table (XLSX)

S3 Table (XLSX)

S4 Table (XLSX)

S1 Video Video presentation of experimental program. (MP4)

## References

[1] Balendra T, Wang CM, Rakesh G. Effectiveness of TLCD on various structural systems. Eng Struct. 1999;21: 291–305. 10.1016/S0141-0296(97)00156-9

[2] Jangid RS, Datta TK. Performance of base isolation systems for asymmetric building subject to random excitation. Eng Struct. 1995;17: 443–454. 10.1016/0141-0296(95)00054-B

[3] Kwok KCS, Samali B. Performance of tuned mass dampers under wind loads. Eng Struct. 1995;17: 655–667. 10.1016/0141-0296(95)00035-6

[4] Tamura Y, Fujii K, Ohtsuki T, Wakahara T, Kohsaka R. Effectiveness of tuned liquid dampers under wind excitation. Eng Struct. 1995;17: 609–621. 10.1016/0141-0296(95)00031-2

[5] Balendra T, Wang CM, Cheong HF. Effectiveness of tuned liquid column dampers for vibration control of towers. Eng Struct. 1995;17: 668–675. 10.1016/0141-0296(95)00036-7

[6] Balendra T, Wang C., Yan N. Control of wind-excited towers by active tuned liquid column damper. Eng Struct. 2001;23: 1054–1067. 10.1016/S0141-0296(01)00015-3

[7] Yalla SK, Kareem A. Optimum Absorber Parameters for Tuned Liquid Column Dampers. J Struct Eng. 2000;126: 906–915. 10.1061/(ASCE)0733-9445(2000)126:8(906)

[8] Das S, Choudhury S. Seismic response control by tuned liquid dampers for low-rise RC frame buildings. Aust J Struct Eng. 2017;18: 135–145. 10.1080/13287982.2017.1351180

[9] Gao H, Kwok KCS, Samali B. Optimization of tuned liquid column dampers. Eng Struct. 1997;19: 476–486. 10.1016/S0141-0296(96)00099-5

[10] Chen J-L, Georgakis CT. Spherical tuned liquid damper for vibration control in wind turbines. J Vib Control. 2015;21: 1875–1885. 10.1177/1077546313495911

[11] Xu YL, Samali B, Kwok KCS. Control of Along‐Wind Response of Structures by Mass and Liquid Dampers. J Eng Mech. 1992;118: 20–39. 10.1061/(ASCE)0733-9399(1992)118:1(20)

[12] Haroun MA, Pires JA, Won AYJ. Suppression of Environmentally-Induced Vibrations in Tall Buildings by Hybrid Liquid Column Dampers. Struct Des Tall Build. 1996;5: 45–54. 10.1002/(SICI)1099-1794(199603)5:1<45::AID-TAL58>3.0.CO;2-F

[13] Huo L-S, Li H-N. Torsionally Coupled Response Control of Structures Using Circular Tuned Liquid Column Dampers.: 11.

[14] Sadek F, Mohraz B, Lew HS. Single- and multiple-tuned liquid column dampers for seismic applications. Earthq Eng Struct Dyn. 1998;27: 439–463. 10.1002/(SICI)1096-9845(199805)27:5<439::AID-EQE730>3.0.CO;2-8

[15] Gao H, Kwok KSC, Samali B. Characteristics of multiple tuned liquid column dampers in suppressing structural vibration. Eng Struct. 1999;21: 316–331. 10.1016/S0141-0296(97)00183-1

[16] Zhu F, Wang J-T, Jin F, Lu L-Q. Real-time hybrid simulation of full-scale tuned liquid column dampers to control multi-order modal responses of structures. Eng Struct. 2017;138: 74–90. 10.1016/j.engstruct.2017.02.004

[17] Yalla SK, Kareem A, Kantor JC. Semi-active tuned liquid column dampers for vibration control of structures. Eng Struct. 2001;23: 1469–1479. 10.1016/S0141-0296(01)00047-5

[18] Sonmez E, Nagarajaiah S, Sun C, Basu B. A study on semi-active Tuned Liquid Column Dampers (sTLCDs) for structural response reduction under random excitations. J Sound Vib. 2016;362: 1–15. 10.1016/j.jsv.2015.09.020

[19] Wang JY, Ni YQ, Ko JM, Spencer BF. Magneto-rheological tuned liquid column dampers (MR-TLCDs) for vibration mitigation of tall buildings: modelling and analysis of open-loop control. Comput Struct. 2005;83: 2023–2034. 10.1016/j.compstruc.2005.03.011

[20] Ni YQ, Ying ZG, Wang JY, Ko JM, Spencer BF. Stochastic optimal control of wind-excited tall buildings using semi-active MR-TLCDs. Probabilistic Eng Mech. 2004;19: 269–277. 10.1016/j.probengmech.2004.02.010

[21] Park B, Lee Y, Park M, Ju YK. Vibration control of a structure by a tuned liquid column damper with embossments. Eng Struct. 2018;168: 290–299. 10.1016/j.engstruct.2018.04.074

[22] Pandey DK, Sharma MK, Mishra SK. A compliant tuned liquid damper for controlling seismic vibration of short period structures. Mech Syst Signal Process. 2019;132: 405–428. 10.1016/j.ymssp.2019.07.002

[23] Koo J-H, Jang D-D, Usman M, Jung H-J. A feasibility study on smart base isolation systems using magneto-rheological elastomers. Struct Eng Mech. 2009;32: 755–770. 10.12989/sem.2009.32.6.755

[24] Usman M, Jang D-D, Kim I-H, Jung H-J, Koo J-H. Dynamic Testing and Modeling of Magneto-Rheological Elastomers Volume 1: Active Materials, Mechanics and Behavior; Modeling, Simulation and Control. Oxnard, California, USA: ASME; 2009 pp. 495–500. 10.1115/SMASIS2009-1348

[25] Ying ZG, Ni YQ, Ko JM. Semi-active optimal control of linearized systems with multi-degree of freedom and application. J Sound Vib. 2005;279: 373–388. 10.1016/j.jsv.2003.11.004

[26] Hochrainer MJ, Ziegler F. Control of tall building vibrations by sealed tuned liquid column dampers. Struct Control Health Monit. 2006;13: 980–1002. 10.1002/stc.90

[27] Khalid B, Ziegler F. A novel aseismic foundation system for multipurpose asymmetric buildings. Arch Appl Mech. 2012;82: 1423–1437. 10.1007/s00419-012-0667-8

[28] Di Matteo A, Furtmüller T, Adam C, Pirrotta A. Optimal design of tuned liquid column dampers for seismic response control of base-isolated structures. Acta Mech. 2018;229: 437–454. 10.1007/s00707-017-1980-7

[29] Khan BL, Azeem M, Usman M, Farooq SH, Hanif A, Fawad M. Effect of near and far Field Earthquakes on performance of various base isolation systems. Procedia Struct Integr. 2019;18: 108–118. 10.1016/j.prostr.2019.08.145

[30] Al-Saif KA, Aldakkan KA, Foda MA. Modified liquid column damper for vibration control of structures. Int J Mech Sci. 2011;53: 505–512. 10.1016/j.ijmecsci.2011.04.007

[31] Gur S, Roy K, Mishra SK. Tuned liquid column ball damper for seismic vibration control: TUNED LIQUID COLUMN BALL DAMPER. Struct Control Health Monit. 2015;22: 1325–1342. 10.1002/stc.1740

[32] Gupta A, Kakulate M, Jopale A. Spring Loaded Liquid Column Ball Damper for Vibration Control with Forced Vibration. 2017;5: 7.

[33] Pandey DK, Mishra SK. Moving orifice circular liquid column damper for controlling torsionally coupled vibration. J Fluids Struct. 2018;82: 357–374. 10.1016/j.jfluidstructs.2018.07.015

[34] Inamdar NJ. Educational Shaking Table Modules for Earthquake Engineering, Masters Dissertation, University of Texas, 2010.

[35] Mišljen P, Matijevi´c M, Despotovi´c Ž. Modeling and Control of Bulk Material Flow on the Electromagnetic Vibratory Feeder. Automatika. 2016;57: 936–947. 10.7305/automatika.2017.03.1766

[36] Ali A, Sandhu T, Usman M. Ambient Vibration Testing of a Pedestrian Bridge Using Low-Cost Accelerometers for SHM Applications. Smart Cities. 2019;2: 20–30. 10.3390/smartcities2010002

